# Addressing an opportune time to revise the Philippine Comprehensive Dangerous Drugs Act of 2002

**DOI:** 10.1016/j.lanwpc.2023.100857

**Published:** 2023-07-22

**Authors:** Rowalt Alibudbud

**Affiliations:** Department of Sociology and Behavioral Sciences, College of Liberal Arts, De La Salle University, 2401 Taft Avenue, Manila City, 1004, Philippines

Smith et al.^1^ emphasized the imperative of implementing evidence-based public health programs and advancing social justice in drug rehabilitation and treatment within the Philippines. The authors also underlined the necessity of reforming the two-decades-old Comprehensive Dangerous Drugs Act.^1^ This stance is consistent with the position of local and international advocates, students, researchers, and academics who have called upon the Philippine government to revise the Act in accordance with scientific evidence and human rights principles.^2^ Therefore, it is an opportune time to revise the Act.

First, the Act provides severe penalties for illicit drug-related activities, including the apprehension of people who used drugs (PWUDs), including a minimum six-month rehabilitation period in a government facility for first-time offenders found to be using illicit drugs.^3^ However, limited evidence substantiates the advantages of compulsory drug treatment, and certain studies indicated potential harms associated with such approaches.^4^ Considering the potential for human rights violations within compulsory settings, it has been recommended that policymakers prioritize non-compulsory and voluntary treatment options.^4^ While provisions for voluntary treatment options are present within the Act, the imposition of involuntary treatment should be repealed.

Second, substance use disorders are chronic relapsing conditions.^5^ Nonetheless, the Act imposes prison sentences ranging from six to twelve years for individuals arrested for a second offence of illicit drug use.^3^ Therefore, it is necessary to enhance mental health support in the context of voluntary treatment and rehabilitation.

Third, PWUDs can be subjected to fines ranging from PhP 50,000 to PhP 200,000 (approximately USD 1000–4000).^3^ Since reports indicate that economically disadvantaged Filipinos have been disproportionately affected by the War on Drugs,^1^^,^^2^ it is essential to eliminate financial penalties to prevent exacerbating their economic hardships.

Fourth, the Act establishes the Dangerous Drug Board (DDB) as the policymaking and strategy-formulating body for drug prevention and control in the Philippines.^3^ Given the necessity for local evidence about drug treatment and rehabilitation,^2^ it is crucial to expand the DDB's membership to the Department of Science and Technology, the agency responsible for science and technology-related projects and policies.

Fifth, in light of the reported human rights violations during the Philippine War on Drugs,^2^ the DDB's membership can be extended to the Philippine Commission on Human Rights. Moreover, the inclusion of observers from international human rights organizations, such as the United Nations OHCHR and Human Rights Watch, would help ensure that policies are better informed and adhere to human rights principles.

## Declaration of interests

I declare no competing interests.
